# Axl acts as a tumor suppressor by regulating LIGHT expression in T lymphoma

**DOI:** 10.18632/oncotarget.15830

**Published:** 2017-03-02

**Authors:** Eun-Hee Lee, Eun-Mi Kim, Kon-Ji Young, A-Reum Park, Ha-Rim Choi, Hwa-Youn Lee, Su-Man Kim, Byung Yeoup Chung, Chul-Hong Park, Hyo Jin Choi, Young-Hyeh Ko, Hyoung-Woo Bai, Hyung-Sik Kang

**Affiliations:** ^1^ Research Division for Biotechnology, Advanced Radiation Technology Institute (ARTI), Korea Atomic Energy Institute (KAERI), Jeongeup-si, Jeollabuk-do 580-185, Republic of Korea; ^2^ Predictive Model Research Center, Korea Institute of Toxicology, Yuseong-gu, Daejeon, 34114, Republic of Korea; ^3^ School of Biological Sciences and Technology, Chonnam National University, Buk-gu, Gwangju 500-757, Republic of Korea; ^4^ Department of Nursing, Nambu University, Gwangsan-gu, Gwangju 506-706, Republic of Korea; ^5^ Medical Device Development Center, Daegu-Gyeongbuk Medical Innovation Foundation, Dong-gu, Daegu 701-310, Republic of Korea; ^6^ Department of Pathology, Samsung Medical Center, Sungkyunkwan University School of Medicine, Gangnam-gu, Seoul 135-710, Republic of Korea

**Keywords:** Axl receptor tyrosine kinase, LIGHT, T lymphoma, anti-tumorigenicity, oncogene, Immunology and Microbiology Section, Immune response, Immunity

## Abstract

Axl is an oncogenic receptor tyrosine kinase that plays a role in many cancers. LIGHT (Lymphotoxin-related inducible ligand that competes for glycoprotein D binding to herpesvirus entry mediator on T cells) is a ligand that induces robust anti-tumor immunity by enhancing the recruitment and activation of effector immune cells at tumor sites. We observed that mouse EL4 and human Jurkat T lymphoma cells that stably overexpressed Axl also showed high expression of LIGHT. When Jurkat-Axl cells were treated with Gas6, a ligand for Axl, LIGHT expression was upregulated through activation of the PI3K/AKT signaling pathway and transcriptional induction by Sp1. The lytic activity of cytotoxic T lymphocytes and natural killer cells was enhanced by EL4-Axl cells. In addition, tumor volume and growth were markedly reduced due to enhanced apoptotic cell death in EL4-Axl tumor-bearing mice as compared to control mice. We also observed upregulated expression of CCL5 and its receptor, CCR5, and enhanced intratumoral infiltration of cytotoxic T lymphocytes and natural killer cells in EL4-Axl-bearing mice as compared to mock controls. These data strongly suggested that Axl exerts novel tumor suppressor effects by inducing upregulation of LIGHT in the tumor microenvironment of T lymphoma.

## INTRODUCTION

The mammalian TAM receptor tyrosine kinase family consists of three receptors, namely, Tyro-3, Axl and Mer [1]. Axl is a transmembrane receptor that is ubiquitously expressed in epithelial and hematopoietic cells [2–4]. Axl was originally described as an oncogene isolated from chronic myeloid leukemia patients [5]. Axl is overexpressed in a variety of tumor cells and several types of human cancers [6–9]. Gas6 (Growth-arrest-specific protein 6) and protein S are the two known biological ligands for Axl, with Gas6 demonstrating a higher affinity for Axl than protein S [10]. Furthermore, the Gas6/Axl signaling pathway regulates many cellular processes, such as cell proliferation, survival, migration and adhesion; blood clot stabilization; inflammation; cytokine release; and phagocytosis of apoptotic cells [11]. The Gas6/Axl pathway is also associated with phosphatidylinositol 3-kinase (PI3K)/AKT, extracellular signal-regulated kinase (ERK) and nuclear factor kappa B (NF-κB) signaling in various cell types [12, 13]. The Gas6/Axl signaling pathway suppresses apoptotic cell death through the inhibition of pro-apoptotic caspase 3 and phosphorylation of NF-κB [12]. Also, ERK mediates Gas6-induced human prostate cancer cell proliferation suggesting that the Gas6/Axl signaling pathway may be involved in the tumor evasion mechanism by suppressing the pro-apoptotic effects of numerous chemotherapeutics in many human cancers [13]. Interestingly, Axl expression was rare in lymphocytic leukemia compared to myeloid leukemia {19 of 54 cases (35%)[14]} with only 1 of 45 (2.2%) of lymphoid leukemia patients expressing Axl mRNA [2, 15, 16]. A recent report demonstrated that Axl was constitutively phosphorylated in the primary B cells derived from chronic lymphocytic leukemia (CLL) patients and its expression correlated with the proliferation or apoptosis rate of CLL [17]. However, Axl transcripts were especially absent in T lymphoma patients.

Light (Lymphotoxin-related inducible ligand that competes for glycoprotein D binding to herpesvirus entry mediator (HVEM) on T cells) is a TNF superfamily ligand known as TNFSF14 that modulates T cell immune responses by signaling through HVEM or lymphotoxin β receptor (LTβR) [18, 19]. The two receptors for LIGHT, namely, LTβR and HVEM, are expressed differentially in various cell types. LTβR is expressed predominantly on stromal and epithelial cells [20–22]. It is also expressed on dendritic cells (DCs) and monocytes, but absent in lymphocytes [23]. In contrast, HVEM is predominantly expressed in hematopoietic cells [24, 25] and other cell types including primary epithelial cells, breast cancer cell lines and pancreatic β cells [26–28]. LIGHT is involved in regulation of apoptosis [29], control of the immune response by enhancing T cell proliferation and cytokine secretion [24, 30, 31] and induction of DC maturation [32]. Furthermore, LIGHT demonstrates potent anti-tumor activity by inducing massive infiltration of naive T lymphocytes, which correlates with upregulation of both chemokine production and expression of adhesion molecules [33]. However, the role of LIGHT expression in context of Axl function is unknown.

Whereas oncogenic role of Axl has been identified in various cancers, LIGHT performed as a tumor suppressor in LIGHT-overexpressing A20 B lymphoma cells [34]. However, our data showed that mRNA and protein expression of Axl and LIGHT was lowly expressed in EL4 T lymphoma cells. Further, LIGHT mRNA transcripts were suppressed in the thymus of Axl^−/−^ mice. Since these data were inconsistent with their previously demonstrated contrasting roles, we investigated if Axl perfomed a novel tumor suppressor role in T lymphoma by positively regulating LIGHT expression.

## RESULTS

### Axl-induced upregulation of LIGHT expression

**Figure 1:**
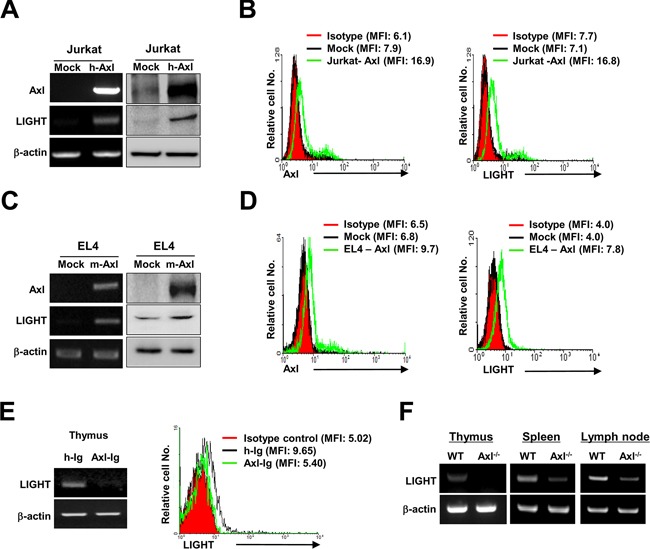
Axl-induced upregulation of LIGHT expression in human and mouse T lymphoma The expression levels of Axl and LIGHT were determined by RT-PCR (A and C, left), western blotting (A and C, right panel) and flow cytometry analysis (B and D) in transfectants stably overexpressing Axl in human Jurkat (Jurkat-Axl) and mouse T lymphoma cells (EL4-Axl), respectively. (E) The expression of LIGHT was determined by RT-PCR and flow cytometry analysis in the thymuses isolated from WT mice that were intraperitoneally injected with Axl-Ig (100μg/mouse) or control h-Ig for 3 weeks (n = 5). The values in parentheses on histograms of B, D and E are mean fluorescence intensity (MFI) for Axl and LIGHT staining. (F) The gene expression of LIGHT was assessed by RT-PCR in the thymus, spleen and lymph node isolated from WT and Axl^−/−^ mice (n = 5). Data are representative of at least five independent experiments

Since Axl and LIGHT had contrasting roles in tumor immunity, we expected contrasting expression patterns in the T lymphoma cells. However, RT-PCR and western blot analysis showed that the expression levels of both Axl and LIGHT were elevated in human Jurkat T lymphoma cells that stably overexpressed Axl compared to the mock control (Figure 1A). Further, enhanced cell surface expression of Axl and LIGHT was observed in Jurkat-Axl cells as determined by flow cytometry analysis (MFI for Axl: 7.9 in mock *vs*. 16.9 in Jurkat-Axl; MFI for LIGHT: 7.1 in mock *vs*. 16.8 in Jurkat-Axl) (Figure 1B). Similar expression pattern was also observed in mouse EL4-Axl T lymphoma cells (Figures 1C and 1D), implying that LIGHT expression may be regulated by Axl. To further determine if Axl signaling regulated LIGHT expression, we assessed LIGHT expression in WT mice that were administered with a neutralizing fusion protein specific for Axl (Axl-Ig). We observed that inhibition of Axl signaling with Axl-Ig suppressed expression of LIGHT mRNA and protein in WT mice (Figure 1E). Consistent with these data, LIGHT mRNA expression was lower in the thymus, spleen and lymph node of Axl^−/−^ mice compared to the WT mice (Figure 1F). These results suggested that Axl upregulated the expression of LIGHT in T lymphoma.

### Axl-mediated upregulation of LIGHT expression via the PI3K/AKT signaling pathway

**Figure 2:**
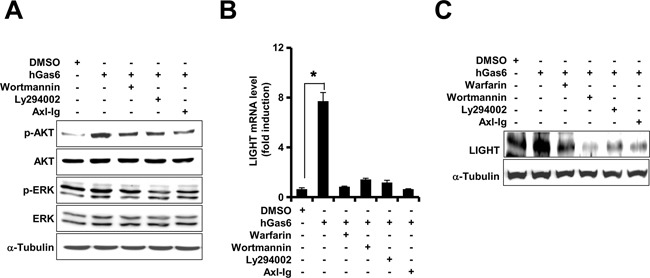
PI3K/AKT signaling pathway is necessary for Axl-induced upregulation of LIGHT (**A**) After pretreatment with 1μM of Wortmannin, 20μM of LY294002 or1μg/ml of Axl-Ig for 1h, Jurkat-Axl cells were treated with 1μg/ml of human Gas6 (hGas6) for 5 min in serum-free RPMI medium. The levels of phosphorylation were evaluated by western blotting using the indicated antibodies. (**B** and **C**) After the pretreatment of Jurkat-Axl cells with the same doses of the indicated signal blockers for 1h as described above, the cells were stimulated with hGas6. The gene and protein expression levels of LIGHT were analyzed after 6h or 24h by qRT-PCR (B) and western blot analysis (C), respectively. The data in B are shown as the mean ± SEM (*, P < 0.05; **, P < 0.01, ***, P < 0.001). Data are representative of five independent experiments

PI3K/AKT and ERK are downstream targets in the Axl signal transduction pathway [12, 13]. To analyze the activation status of these downstream targets in Axl-mediated upregulation of LIGHT expression, Jurkat-Axl cells were stimulated with human Gas6 (hGas6) in presence of Axl-Ig or PI3K/AKT inhibitors, Wortmannin and LY294002. As shown in Figure 2A, the phosphorylation of AKT increased markedly within 5 min after stimulation with rhGas6 and was diminished by treatment with Wortmannin, LY294002 and Axl-Ig. However, the levels of ERK phosphorylation were comparable between the treatment groups. Further, treatment with Wortmannin, LY294002 and Axl-Ig abolished the expression of LIGHT mRNA and protein, which was upregulated by rhGas6 (Figure 2). Together, these data suggested that the PI3K/AKT signaling pathway was involved in the upregulation of LIGHT by Axl.

### Transcriptional activation of Sp1 in Axl-induced LIGHT expression

**Figure 3:**
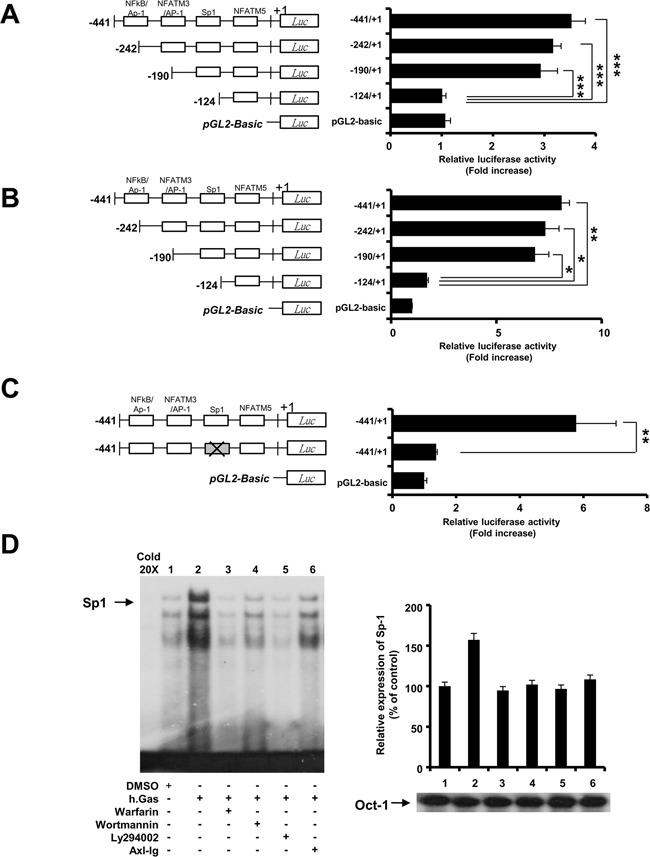
Transcriptional activation of Sp1 regulates Axl-mediated LIGHT expression. (**A and B**) Jurkat and 293T cells were co-transfected with plasmids as described in the Materials and Methods. Luciferase activity was measured in Jurkat and 293T cells stimulated with 1μg/ml of rhGas6 for 24h. (**C**) Site-directed mutagenesis was performed on Sp1 binding site of LIGHT promoter followed by the luciferase assay in 293T cells stimulated with rhGas6. Data are represented as the mean ± SEM from three independent experiments (*, P < 0.05; **, P < 0.01, ***, P < 0.001). (**D**) Jurkat-Axl cells were pretreated with signal blockers as described in Figure 2 followed by assessment of the DNA-binding activity of Sp1 by EMSA after stimulation with 1μg/ml of rhGas6 for 3h. Oct-1 was used as an internal protein loading control. Cold 20x represents a 20-fold excess of unlabeled Sp1 probe for competition analysis.

To identify the transcription factors activated by Axl signaling, we generated deletion mutants in the proximal promoter region of LIGHT and co-transfected transiently with pcDNA3.1-*Axl* into Jurkat and 293T cells. After treating the transfectants with hGas6 for 24h, we observed robust reduction in LIGHT promoter activity for the deletion mutant of the 190 to 124 region whereas the deletion mutant of the 441 to 190 region was unaffected in both Jurkat (A) and 293T (B) cells (Figure 3). To corroborate these observations, we performed the site-directed mutagenesis of Sp1 binding site on the LIGHT promoter and observed that it reduced the relative luciferase activity to basal level in the 293T cells (Figure 3C). This suggested that the transcriptional activation of Sp1 was required for LIGHT expression.

**Figure 4:**
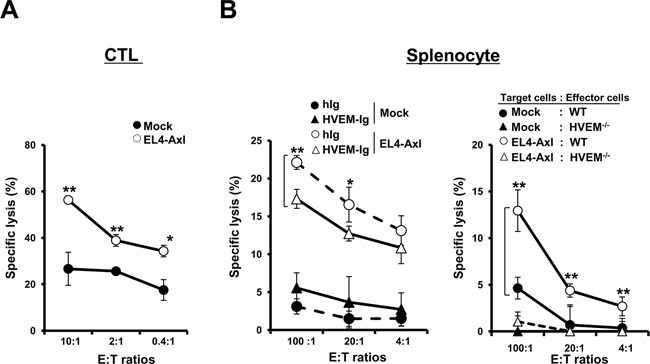
Enhanced CTL and NK activity results from the interaction between HVEM and Axl-induced LIGHT expression **(A)** Purified CD8^+^ CTLs were stimulated with dendritic cells primed by 50μg of EL4 tumor lysates. CTL activity was determined by the lactate dehydrogenase (LDH) assay at the indicated effector:target (E:T) ratios. **(B)** (Left panel) For the preparation of target cells, EL4-Axl cells and mock controls were treated with 500ng/ml of HVEM-Ig or control h-Ig for 24h. NK cytotoxicity was determined by the LDH assay at the indicated E:T ratios using splenocytes isolated from WT mice as effectors. (Right panel) After stimulation of splenocytes isolated from WT and HVEM^−/−^ mice with IL-2 (20ng/ml) for 24h, NK cytotoxicity was measured using EL4-Axl cells or mock controls as target cells. These data are shown as the mean ± SEM (*, P < 0.05; **, P < 0.01, ***, P < 0.001) and are representative of five independent experiments.

**Figure 5:**
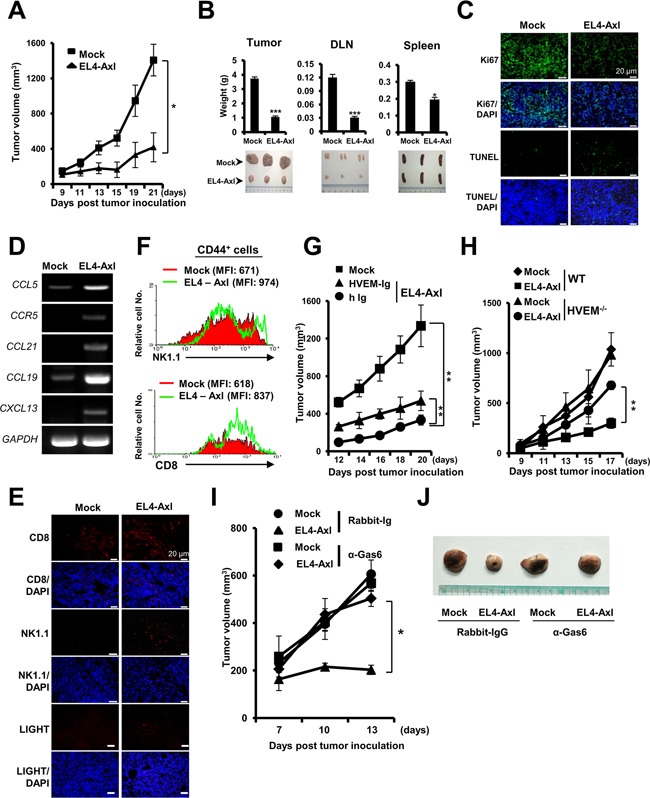
Anti-tumorigenic effect of Axl-induced LIGHT expression in EL4 T lymphoma-bearing mice. WT mice injected with EL4-Axl cells and mock controls were sacrificed at 21 days (n=8) **(A** and **B)** Changes in the volume **(A)**, weight (**B**, left and top) and shape (**B**, left and bottom) of tumors are shown. The weight and shape of the draining lymph node (DLN) and spleen from the indicated mice are shown. Tumor progression-associated molecules were evaluated as follows: **(C and E)** Immunohistofluorescence analysis using the indicated antibodies. **(D)** qRT-PCR using each chemokine-specific primer. **(F)** In the flow cytometry analysis, the activation status of CTLs and NK cells was determined by mean fluorescence intensity of CD8^+^ or NK1.1^+^ cells on gated CD44^+^ cells. **(G** and **H)** Tumor volume of HVEM-Ig-administered EL4-Axl tumor-bearing WT mice and of EL4-Axl tumor-bearing in HVEM^−/−^ mice. **(I** and **J)** Tumor volume and size of the Gas6-deficient EL4-Axl-bearing mice (n=4). The data from A, B, D, G and H are shown as the mean ± SEM (*, P < 0.05; **, P < 0.01, ***, P < 0.001). Data are representative of five independent experiments.

Next, to verify if the transcriptional activity of Sp1 on the LIGHT promoter was regulated by Axl signaling, we performed the electrophoretic mobility shift assay (EMSA) in Jurkat-Axl cells and determined Sp1 binding activity after treatment with Axl signal blockers in the presence of Gas6. We observed that Sp1 binding activity was enhanced by rhGas6 treatment and suppressed by Axl signal blockers (Figure 3C). These results suggested that Sp1 regulated LIGHT gene expression by Axl signaling.

### Axl-overexpressing T lymphoma cells demonstrate enhanced susceptibility to cytotoxic T lymphocytes- and natural killer cells-mediated cytotoxicity

The expression of LIGHT on tumor cells and its interaction with HVEM plays a crucial role in the activation of cytotoxic T lymphocytes (CTLs) and natural killer (NK) cells [33, 35]. To investigate if Axl-mediated LIGHT expression was capable of enhancing the susceptibility of T lymphoma cells to CTLs and NK cells, cytotoxicity assays were performed with CTLs and NK cells from WT mice as effector immune cells and EL4-Axl cells as target cells. We observed that the lytic activity of CTLs was enhanced in EL4-Axl cells compared to the mock controls (Figure 4A). Similarly, NK cytotoxic activity was enhanced in EL4-Axl cells and inhibited by treatment with HVEM-Ig compared to control human Ig (hIg) (Fig. 4B, left panel). This implied that blocking LIGHT signaling reduced the susceptibility of EL4-Axl target cells to NK-mediated lysis. Further, we investigated NK cytotoxic activity in splenocytes isolated from HVEM^−/−^ mice to understand the importance of Axl mediated LIGHT expression. Our data showed that NK activity was significantly inhibited in HVEM^−/−^ mice compared to WT mice (Fig. 4B, right panel). These findings suggested that LIGHT expression induced by Axl signaling enhanced the susceptibility to CTL- and NK-mediated lysis of target cells.

### *In vivo* anti-tumorigenic effects of Axl by upregulating LIGHT expression

LIGHT expression in the tumor microenvironment inhibited tumor formation, growth and progression [33]. To investigate the *in vivo* effect of Axl on tumorigenesis, tumor formation and growth were evaluated in mock and EL4-Axl tumor-bearing mice. The tumor volume was significantly smaller in EL4-Axl-tumor bearing mice than the mock controls (Figure 5A). Further, the weights of the tumors, draining lymph nodes and spleens were remarkably lower in EL4-Axl-bearing mice (Figure 5B). Immunohistofluorescence analysis showed that the distribution of cells that positively stained with the Ki-67 proliferative marker dramatically decreased in the tumor tissue of EL4-Axl-bearing mice (Figure 5C). Moreover, a higher rate of apoptotic cell death was observed in EL4-Axl-bearing mice as evaluated by the TUNEL assay. The chemokine C-C motif ligand 5 (CCL5) and its receptor, CCR5 are critical regulators for the infiltration of immune cells into the tumor microenvironment [36]. We observed that the mRNA expression of CCL5 and CCR5 as well as LTβR-related chemokines, such as CCL21, CCL19 and CXCL13 were substantially enhanced in the tumor tissue of EL4-Axl-bearing mice compared with mock control (Figure 5D). To address whether the increased expression of those chemokines was associated with the infiltration of immune cells into the tumor region, the distribution of CTLs and NK cells was determined by immunohistofluorescence analysis using anti-CD8 and anti-NK1.1 antibodies, respectively. Consistent with the expression of those chemokines, the intratumoral infiltration of CTLs and NK cells was more strongly induced in EL4-Axl-bearing mice than in mock controls (Figure 5E). The expression of LIGHT also increased in the tumor region of EL4-Axl-bearing mice, correlating with the high frequency of CTLs and NK cells. Moreover, flow cytometry showed that the tumor tissue of EL4-Axl-bearing mice had higher percentages of activated CTLs (CD8^+^CD44^+^; MFI, 837) and NK cells (NK1.1^+^CD44^+^; MFI, 974) than in the mock controls (CD8^+^CD44^+^; MFI, 618, NK1.1^+^CD44^+^; MFI, 671) (Figure 5F).

Next, to corroborate the effect of tumor eradication by Axl-induced LIGHT expression, tumor volume was measured in EL4-Axl-tumor bearing WT mice that were administered with HVEM-Ig and the HVEM^−/−^ mice. We observed that the tumor volume was significantly increased in EL4-Axl-tumor bearing WT mice treated with HVEM-Ig (Figure 5G). Furthermore, EL4-Axl-tumor bearing HVEM^−/−^ mice had much larger tumor volumes than EL4-Axl-bearing WT mice (Figure 5H). To analyze the effect of Axl-mediated tumor eradication in Axl ligand-deficient mice, EL4-Axl-bearing mice were injected with rabbit anti-Gas6 polyclonal antibody. The tumors in Gas6-deficient EL4-Axl-tumor bearing mice were much larger in volume and size than those of control rabbit Ig-injected EL4-Axl-tumor bearing mice (Figures 5I and 5J). No differences were observed in tumor volume and size between Gas6-deficient mock-control mice and control rabbit Ig-injected mock-control mice. These data suggested that Axl acted as a tumor suppressor by inducing LIGHT expression in the tumor microenvironment of T lymphoma.

## DISCUSSION

Contrary to previous data that Axl was an oncogene and that LIGHT was a tumor suppressor, we found that the expression patterns of LIGHT and Axl were similar in Axl overexpressing T lymphomas. Further experiments identified a novel tumor suppressor function for Axl in regulating LIGHT expression in T lymphomas. Although Sp1 binding to LIGHT promoter elements had previously been identified [37], the upstream components leading to transcriptional activation of Sp1 were unknown. We found that Axl-induced LIGHT expression was triggered by the activation of Sp1 through the PI3/AKT signaling pathway. Moreover, the expression of LIGHT was triggered by inducing Sp1 transcriptional activation in response to Axl signaling in Jurkat cells, providing evidence that Sp1 activation was required for Axl-induced LIGHT expression in T lymphoma. Recently, the Axl signaling pathway has been identified as a very promising target for cancer therapy [38]. However, our data suggests that Axl act as a tumor suppressor in T lymphoma. This is further supported by recent studies that show increased susceptibility of Axl^−/−^ Mer^−/−^ mice and Gas6^−/−^ mice to azoxymethane/dextran sulfate sodium-induced colitis-associated colorectal cancer compared to WT mice [39, 40]. Although further investigations are necessary to identify the mechanisms in other cancers, our data strongly suggest that suppression of T lymphoma progression is elicited by Axl through the regulation of LIGHT expression.

In our experiments, compared to EL4-Axl cells, the tumor tissues of EL4-Axl-tumor bearing mice demonstrated high mRNA expression of CCL5 and CCR5 (data not shown). Furthermore, the expression of LT#βR-related chemokines, such as CCL21, CCL19 or CXCL13 increased in the EL4-Axl-tumor bearing mice compared to the mock control. These differences in expression of chemokines could be attributed to the *in vivo* interaction between Axl-induced LIGHT on EL4-Axl tumor cells and its receptor, LTβR on the stromal cells, thereby inducing their expression. Furthermore, the increased expression of the chemokines was associated with the infiltration of CTLs and NK cells into the tumor region in EL4-Axl-bearing mice. Several studies have demonstrated that infiltrated CTLs and NK cells express HVEM, which interacts with LIGHT on the tumor cells and thereby induces the activation of CTLs and NK cells [33, 35]. In the present study, the enhanced *in vitro* cytotoxic activity of CTLs or NK cells in the EL4-Axl target cells compared to the controls was reversed by either HVEM-Ig treatment or in HVEM^−/−^ mice. Furthermore, EL4-Axl-bearing mice had a higher number of activated NK cells than the mock controls. These data suggested that LIGHT induced by Axl on EL4 cells enhanced their cytotoxic activity by interacting with HVEM on the CTLs and NK cells that had infiltrated due to enhanced expression of CCR5, CCL5 and other LTβR-related chemokines.

In summary, we show for the first time that Axl demonstrates tumor suppressor function by upregulating the expression of LIGHT through the PI3K/AKT signaling pathway, resulting in anti-tumorigenic effects in T lymphoma. Thus, anti-tumorigenic activity of Axl-induced LIGHT may provide a selective target for tumor type-specific anticancer therapies.

## MATERIALS AND METHODS

### Mice, cell lines, and reagents

Seven week old C57BL/6J female mice were obtained from Joongang Experimental Animal Co. (Seoul, South Korea). Axl^−/−^ and HVEM^−/−^ mice were kindly provided by Dr. Greg Lemke from Salk Institute, CA, and Dr. Fu YX from University of Chicago, IL, USA, respectively. The mice were housed in a specific pathogen-free environment and the animal experiments were performed in accordance with the guidelines of Chonnam National University and Korea Atomic Energy Research Institute. EL4 and Jurkat cells were obtained from the American Type Culture Collection (ATCC, Manassas, VA, USA) and maintained in RPMI 1640 medium supplemented with 10% FBS at 37°C in 5% CO_2_ incubator. The IL-2, IL-4, IL-7, IL-15 and GM-CSF cytokines were purchased from Peprotech Inc. (Rocky Hill, NJ, USA). LY294002 and wortmannin were obtained from Cell Signaling Technology (Beverly, MA, USA) and warfarin from Sigma (St. Louis, MO, USA). Human-Ig and Axl-Ig were purchased from R&D System (Minneapolis, MN, USA) and HVEM-Ig was supplied by Dr. Fu YX.

### Antibodies

Western blot, flow cytometry and immunohistofluorescense analysis were conducted using the following antibodies: anti-Axl (Santa Cruz Biotechnology, Santa Cruz, CA, USA); anti-LIGHT and anti-Ki67 (Abcam, Cambridge, MA, USA); anti-AKT, anti-ERK, anti-p-AKT and anti-p-ERK (Cell Signaling Technology, Beverly, MA, USA); FITC-anti-CD8, FITC-anti-NK1.1 and PE-anti-CD44 (BD pharmningen, San Diego, CA, USA).

### Flow cytometry analysis

The surface expression of Axl and LIGHT on stable transfectants was determined by flow cytometry analysis using FACSCalibur after staining with the individual unlabeled primary and anti-goat-PE or anti-rat-Alexa Fluor® 555 secondary antibodies. The results were analyzed by the CellQuest software (BD Bioscience, San Diego, CA, USA).

### Stable transfectants that overexpress Axl in EL4 and Jurkat T lymphoma cells and produce biologically active Gas6 in 293T cells

Full-length cDNA sequences encoding mouse *Axl* (NM_009465.4) and human *AXL* (NM_021913) were cloned into pcDNA3.1 (Invitrogen, Carlsbad, CA, USA) and transfected into EL4 and Jurkat cells using the Amaxa Nucleofector device (Amaxa Inc., Gaithersburg, MD, USA). Stable transfectants with high expression of Axl were selected with 1.0 mg/ml G418 (Sigma) and were further expanded after single-cell cloning by limit dilution. To obtain stable transfectants expressing biologically active human and mouse Gas6, full-length cDNA sequences encoding human *GAS6* (NM_000820.3) and mouse *Gas6* (NM_019521.2) were cloned into pcDNA3.1 and transfected into 293T cells using Lipofectamine (Invitrogen). The stable transfectants were generated as described above. The culture supernatants were collected after incubation of the stable transfectants in serum-free medium containing vitamin K (1μg/ml) for 48h and concentrated using Amicon 8050 ultrafiltration cell (Millipore, Bedford, MA, USA) [41].

### Semiquantitative RT-PCR, quantitative real-time PCR and Western blot analysis

Semiquantitative RT-PCR, quantitative real-time PCR and western blot analysis were carried out as described previously [7]. The primers used for the PCR are listed in Supplementary Table 1.

### Deletion mutants of human LIGHT promoter, site-directed mutagenesis of Sp1 and luciferase reporter assay

The deletion constructs of LIGHT promoter were generated and luciferase assay was performed as described previously [37]. Site-directed mutagenesis of Sp1 on LIGHT promoter was performed on plasmid encoding light promoter -441/+1 in PGL2-basic vector using a QuikChange kit (Stratagene, La Jolla, CA) according to manufacturer’s instruction. The list of primers for deletion constructs of LIGHT promoter and site-directed mutagenesis of Sp1 is listed in Supplementary Table 1. Jurkat and 293T cells were co-transfected with the pGL2-luciferase vector containing LIGHT promoter and the pcDNA3.1 expressing vector encoding Axl. After stimulation of the cells with 1g/ml of human Gas6 (hGas6) for 24h, the luciferase activity was measured and the transfection efficiency was normalized by β-galactosidase activity.

### Electrophoretic mobility shift assay (EMSA)

Nuclear extracts were prepared as described previously [37]. Briefly, 10μg of nuclear extracts was incubated with the [γ-^32^P] dATP-labeled double-stranded oligonucleotide containing Sp1 or OCT-1 binding motifs for 30 min at room temperature. The reaction products were electrophoresed on a 6 % polyacrylamide gel and analyzed by autoradiography. Nucleotide sequences of the oligonucleotides were as follows: SP1, 5′- ATT CGA TCG GGG CGG GGC GAG C-3′ and 5′- GCT CGC CCC GCC CCG ATC GAA T -3′; OCT-1, 5′-TGT CGA ATG CAA ATC ACT AGA A-3′ and 5′- TTC TAG TGA TTT GCA TTC GAC A-3′.

### Cytotoxicity assay of CTLs or NK cells

The cytotoxic activity of CTLs or NK cells was measured by lactate dehydrogenase (LDH)-release assay according to the manufacturer’s instruction (Promega, Madison, WI, USA). Briefly, CD8^+^ T cells were isolated by immunomagnetic selection using microbead conjugated with anti-CD8 in the magnetic field of the vario MACS (Miltenyi Biotec, Bergisch Gladbach, Germany). For the differentiation of monocytes into dendritic cells (DC), monocytes isolated from the bone marrow cells of WT mice were cultured in RPMI 1640 medium supplemented with 10% FBS, 20ng/ml of GM-CSF and IL-4 for 6 days [32]. After priming the differentiated DC with the lysates of EL4 cells (50μg/1×10^6^ cells) for 24h at 37°C, the DC (5×10^5^) were co-cultured with the purified CD8^+^ T cells (1×10^7^) in the presence of IL-7 (10ng/ml) and IL-15 (20ng/ml) for 3 days. The CTLs were then harvested and their cytotoxic activity against EL4-Axl or mock controls was measured by LDH assay. For NK cytotoxicity assay, total splenocytes of HVEM^−/−^ or WT mice stimulated with 20ng/ml of IL-2 were co-cultured for 4h with EL4-Axl or mock controls treated with HVEM-Ig or h-Ig for 24h. The resulting data were presented as the percentage of specific lysis based on the formula: percent specific lysis = (experimental release - spontaneous release) / (maximum release − spontaneous release) × 100.

### Measurement of tumor volume

WT mice were injected subcutaneously with 5×10^5^ EL4 cells on day 0. Seven days later, the mice were administered intratumorally with 100 μg of HVEM-Ig or h-Ig and tumor volumes were measured using external caliper every other day for 2 weeks. For the generation of Gas6-deficient mice, the purified rabbit anti-mouse Gas6 (200μg/dose) was injected three times per week for the duration of experiment. The tumor volumes were calculated by the following modified ellipsoidal formula [42]: Tumor volume = (Length× Width× Height)×Pi/6.

### Histological analysis and immunohistofluorescence

Tumor tissues embedded in OCT compound (DAKO, Carpinteria, CA, USA) were rapidly frozen in liquid nitrogen and cut using Cryostat Leica CM 3050S microcryotome (Leica, Wetzlar, Germany). The tissue sections were fixed with 16% formaldehyde and stained with hematoxylin and eosin as described previously [43]. For evaluation of *in vivo* cell proliferation, the sections were incubated with anti-Ki67 and then stained with Alexa 488-conjugated secondary antibody (Abcam, Cambridge, MA, USA). After counterstaining the sections with DAPI (4, 6-diamidino-2-phenylindole), images were captured with a Olympus IX71 fluorescence microscope (Olympus, Tokyo, Japan). For analysis of apoptosis in the sections, terminal dUTP nick-end labeling (TUNEL) assay was performed using the Promega DeadEnd™ according to manufacturer’s instructions.

### Statistical analysis

The statistical differences were evaluated using one-way ANOVA and Student *t* test. The results were considered statistically significant when *P* values were <0.05. All experiments were performed at least five times independently.

## SUPPLEMENTARY MATERIALS TABLE


