# Supplementary material for: Axl acts as a tumor suppressor by regulating LIGHT expression in T lymphoma
Source: Oncotarget. 2017 Mar 2;8(13):20645–55. doi: 10.18632/oncotarget.15830 (PMC5400533; doi:10.18632/oncotarget.15830)
Supplement: Supplementary file 1 [file oncotarget-08-20645-s001.pdf]

Axl acts as a tumor suppressor by regulating LIGHT expression in T lymphoma

SUPPLEMENTARY TABLE

Supplementary Table 1: Primers used in RT-PCR, real-time PCR and cloning

Primers for RT-PCR		
**Gene**	**Sense (5'-3')**	**Antisense (5'-3')**
mouse Axl	CCC AGA ACC TGT GGT CAT CT	ACC GAG ACA TCA GGG CAT AC
human Axl	CCC AGC ACC TGT GGT CAT CT	ACC GCG ACA TCA AGG CAT AC
mouse LIGHT	GCA TCA ACG TCT TGG AGA CA	ATA CGT CAA GCC CCT CAA GA
human LIGHT	ACA TCT ACT CCA AGG TGC AGCTGG GCG G	CCG AAT TCT CAC ACC ATG AAA GCC CCG AAG TA
β-actin	GTG GGG CGC CCC AGG CAC CA	CTC CTT AAT GTC ACG CAC GAT TTC
**Primers for Real-time PCR**		
**Gene**	**Sense (5'-3')**	**Antisense (5'-3')**
LIGHT	CGT CCG TGT GCT GGA TGA	ACC ATG AAA GCC CCG AAG TA
CCL5	TCT CTG CAG CTG CCC TCA CC	CT TGA ACC CAC TTC TTC TC
CCR5	GAC ATC CGT TCC CCC TAC AAG	TCA CGC TCT TCA GCT TTT TGC AG
**Primers for cloning of deletion constructs of LIGHT promoter**		
**Gene**	**Sense (5'-3')**	**Antisense (5'-3')**
LIGHT(441)-luc	CTA CTC GAG CTT GTC TCT CTGGCT CCA CCA G	CCG AAG CTT GCC CAA GGT GTC TGGAGC AGG GCT GAC ACG C
LIGHT(242)-luc	CTA CTC GAG GAA GCC TCT GGAAAG TGT GAG A	CCG AAG CTT GCC CAA GGT GTC TGGAGC AGG GCT GAC ACG C
LIGHT(190)-luc	CTA CTC GAG GAC CGG GGGCGG CGG GTA CCG G	CCG AAG CTT GCC CAA GGT GTC TGGAGC AGG GCT GAC ACG C
LIGHT(124)-luc	CTA CTC GAG CTC TAA AGG CGGCCC ACG GGT G	CCG AAG CTT GCC CAA GGT GTC TGGAGC AGG GCT GAC ACG C
**Primers for cloning of site directed mutagenesis of Sp1**		
Mutant Sp1 (-193/-168)	GCC AAG ACC GTT GCG GCG GGT ACC	GGT ACC CGC CGC AAC GGT CTT GGC
